# Burden of osteoarthritis in China, 1990–2019: findings from the Global Burden of Disease Study 2019

**DOI:** 10.1007/s10067-024-06885-9

**Published:** 2024-01-30

**Authors:** Hongying Li, Weisi Kong, Yan Liang, Huixin Sun

**Affiliations:** 1 Department of Rheumatology, The First Affiliated Hospital of Harbin Medical University, Harbin City, Heilongjiang Province People’s Republic of China; 2 Institute of Cancer Prevention and Treatment, Harbin Medical University, No. 6 Baojian Road, Nangang District, Harbin City, Heilongjiang Province People’s Republic of China

**Keywords:** China, Disability-adjusted life years, Incidence, Osteoarthritis

## Abstract

This study aimed to report the most current data on the incidence and disability-adjusted life years (DALY) associated with osteoarthritis in China from 1990 to 2019. Publicly available modelled data from Global Burden of Disease Study (GBD) 2019 were used. The incidence and DALY, due to osteoarthritis in China, stratified by sex, trends of associated risk factors, assess the age, period, and cohort effects on the long-term trends of osteoarthritis incidence and DALY in China from 1990 to 2019. We found that the age-standardized incidence and DALY rates of osteoarthritis in China are higher than the average levels in Asia, Africa, and Oceania. In 2019, the number of cases of osteoarthritis in China was 10,681,311, an increase of 132.66% compared with 1990. the DALY of osteoarthritis in China was 4,724,885 person-years, which was 159.70% higher than that in 1990. In 2019, the incidence and DALY rates of osteoarthritis in China was 750.96/100,000,332.19/100,000. High body-mass as risk factors for osteoarthritis DALY with the population attributable proportion (PAF) increasing steadily from 1990 to 2019. The incidence and DALY rates of three types of osteoarthritis from high to low are osteoarthritis knee, osteoarthritis hand, and osteoarthritis hip. Age–period–cohort model showed that the incidence rate of osteoarthritis in China shows a trend of increasing first and then decreasing with age; concurrently, the DALY rate of osteoarthritis in China increased with age. For the period effect, we found that the period rate ratio (*RR*) of osteoarthritis incidence and DALY rates kept increasing in the cohort born before 2005–2009, and then, it was gradually reduced by year of birth in the cohort born after 2005–2009. As for cohort effect, the cohort *RR* of incidence rate of osteoarthritis almost has no change, while the cohort *RR* of DALY rate of osteoarthritis kept increasing from 1990 to 2019. The burden and impact of osteoarthritis in China are substantial and are increasing. Adopting suitable control and preventive community measures to reduce modifiable risk factors is needed to reduce the current and future burden of osteoarthritis in China.

**Table:** 

**Key Points** • *This paper analyzes the disease burden of osteoarthritis in China for the first time and discusses the influence on the disease burden of osteoarthritis from the perspectives of age, period, and cohort*.

**Key Points**

• *This paper analyzes the disease burden of osteoarthritis in China for the first time and discusses the influence on the disease burden of osteoarthritis from the perspectives of age, period, and cohort*.

## Introduction

Osteoarthritis is a common disease in the middle-aged and elderly [1]. Osteoarthritis patients show pain and stiffness of knee, hip, hand, and other joints in the early stage of the disease [2, 3]. With the progress of the disease, severe patients may have limited joint activity or even deformity years later [4, 5]. Systemic conditions in patients with osteoarthritis are probably due to the mild but long-term inflammation that patients present [6]. Global trends showed a 114.5% increase in years lived with disability due to osteoarthritis from 1990 to 2019 [7, 8]. However, there is no nonsurgical intervention that can prevent, halt, or even delay osteoarthritis progression. Moreover, available medications, such as nonsteroidal anti-inflammatory drugs, have been associated with a clinically relevant 50–100% increase in the risk of myocardial infarction or death from cardiovascular causes [9]. Therefore, the public, healthcare providers, and policymakers should be aware of the heavy burden of osteoarthritis. China has a large population base, behind which an extremely large patient population is mapped.

In recent years, the burden of osteoarthritis has been presented in several review papers based on few national studies, and no detailed information was provided in China. In this study, we analyzed the current situation, the temporal trend from 1990 to 2019, and gender differences; in addition, we analyzed and quantified the age, period, and cohort effects on the secular trends of osteoarthritis incidence and disability-adjusted life years (DALY) in China. Our findings should help us better understand osteoarthritis, evaluate current prevention strategies, plan for nationwide management of the burden imposed by osteoarthritis, and improve health management systems to meet future challenges.

## Method

### Overview

Global Burden of Disease Study (GBD) 2019, conducted by Institute of Health Metrics and Evaluation (IHME), is the largest and most comprehensive effort, to date, to measure epidemiological levels and trends worldwide [10].GBD 2019 provides comprehensive and systematic assessments of age- and sex-specific incidence, prevalence, mortality, years of life lost, years lived with disability, and DALY for 369 diseases and injuries in 204 countries and territories from 1990 to 2019. The reference definition of osteoarthritis in the GBD 2019 was symptomatic osteoarthritis that was radiologically confirmed as Kellgren/Lawrence grades 2–4 and painful for at least 1 month of the past 12 months [11]. The general methodology of GBD 2019 developed by IHME and its main improvements compared with previous cycles have been explained in previous publications [12, 13]. Detailed information about fatal and non-fatal estimates used in GBD 2019 can be found at https://vizhub.healthdata.org/gbd-compare/andhttp://ghdx.healthdata.org/gbd-results-tool [10].

### Date source

IHME systematically reviewed the occurrence and frequency of osteoarthritis in the population between 1990 and 2017 for GBD 2017, which only covered osteoarthritis hip and osteoarthritis knee [14]. However, IHME included osteoarthritis hand for GBD 2019, yielding the most thorough and current data on the burden of osteoarthritis available.

The GBD 2019 database was structured following the International Classification of Diseases, 10th edition. In this study, we used the GBD Results Tool to retrieve osteoarthritis disease burden data for China. “China” was selected as the location; “1990 to 2019” as the years; “number and rate” as metrics; “incidence and DALY” as measures; “male, female, both” as genders; “osteoarthritis” as the cause; “metabolic risks and high body mass index” as the risk factors; “osteoarthritis hip, osteoarthritis knee, and osteoarthritis hand” as three types of osteoarthritis; and “30, 35, 40… 95 + ” as the age group. We reported 95% uncertainty intervals derived from 1000 draws from the posterior distribution of each step in the estimation process per established GBD methods.

### Age–period–cohort (APC)model

The APC model was used to analyze the independent effects of age, period, and cohort of osteoarthritis in China from 1990 to 2019. The expression of the model is as follows:

$${\text{ln}}\left({R}_{ijk}\right)={\text{ln}}\left(\frac{{y}_{ij}}{{n}_{ij}}\right)=u+{\alpha }_{i}+{\beta }_{j}+{\gamma }_{k}+\varepsilon$$

where $i$, $j$, and $k$ represent age, period, and cohort group, respectively; ${R}_{ijk}$ represents the incidence and DALY rates of osteoarthritis of the $k$ th cohort at the $j$ th period in the $i$ th age group; ${\alpha }_{i}$, ${\beta }_{j}$, and ${\gamma }_{k}$ represent the estimated effect values of age, period, and cohort, respectively; $u$ represents the intercept of the regression equation; and $\varepsilon$ represents the random error that obeys the normal distribution.

This study used the R-based APC model analysis toolkit developed by IHME in the USA (http://analysistools.cancer.gov/apc/), to conduct statistical analysis. For APC analysis, this study collates and analyzes the incidence and DALY data of osteoarthritis in China according to an age group of every 5 years. The following functions are estimated in the model: Net drift refers to the overall logarithmic linear trend divided by period and cohort, indicating the overall annual percentage change; local drift refers to the logarithmic linear trend of the period and cohort for each age group, representing the annual percentage change for each age group; age effects (longitudinal age curve) are the longitudinal specific age incidence/DALY rate fitted with the selected cohort as the reference after the adjustment of the period deviation; and the transverse time curve is the transverse specific age specific incidence/DALY rate that is fitted with the selected period as the reference after the adjustment of cohort bias. The cohort (or period rate ratio[*RR*]) represents the relative risk of the cohort (or period) relative to the reference cohort (or period) adjusted for age and nonlinear period (or cohort) effects. By default, the middle age, period, and cohort are used as references in the toolkit. The Wald *c*^2^ test is used to test the estimable function [15]. Statistical analysis is conducted using a bilateral test, with a testing level of *α* = 0.05.

### Statistical analysis

The database uses the IHME Bayesian regression tool DisMod-MR V.2.1 for the incidence and DALY of osteoarthritis. The age-standardized rates were calculated by using the GBD 2019 data for a global age-standard population. To analyze changes in China’s disease burden between 1990 and 2019, the formula used is as follows: Changes = (rate in 2019 − rate in 1990)/rate in 1990 × 100%.

## Results

### Global age-standardized incidence and DALY rates of osteoarthritis

**Fig. 1:**
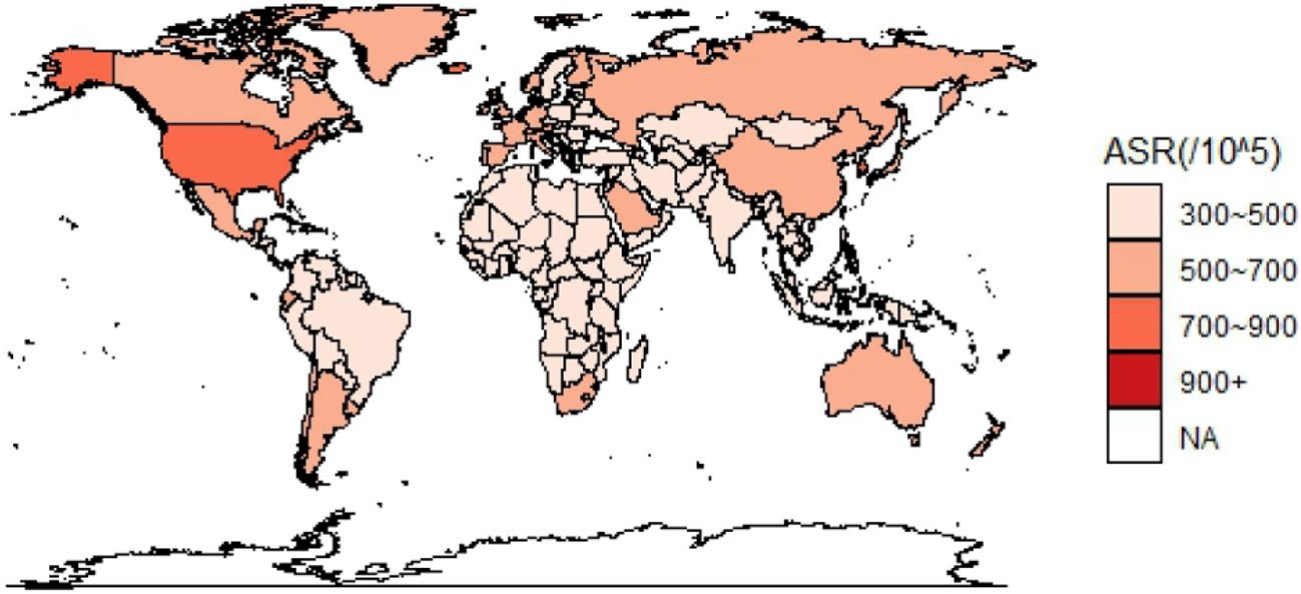
Geographical distribution of incidence rate per 100,000 osteoarthritis worldwide in 2019

**Fig. 2:**
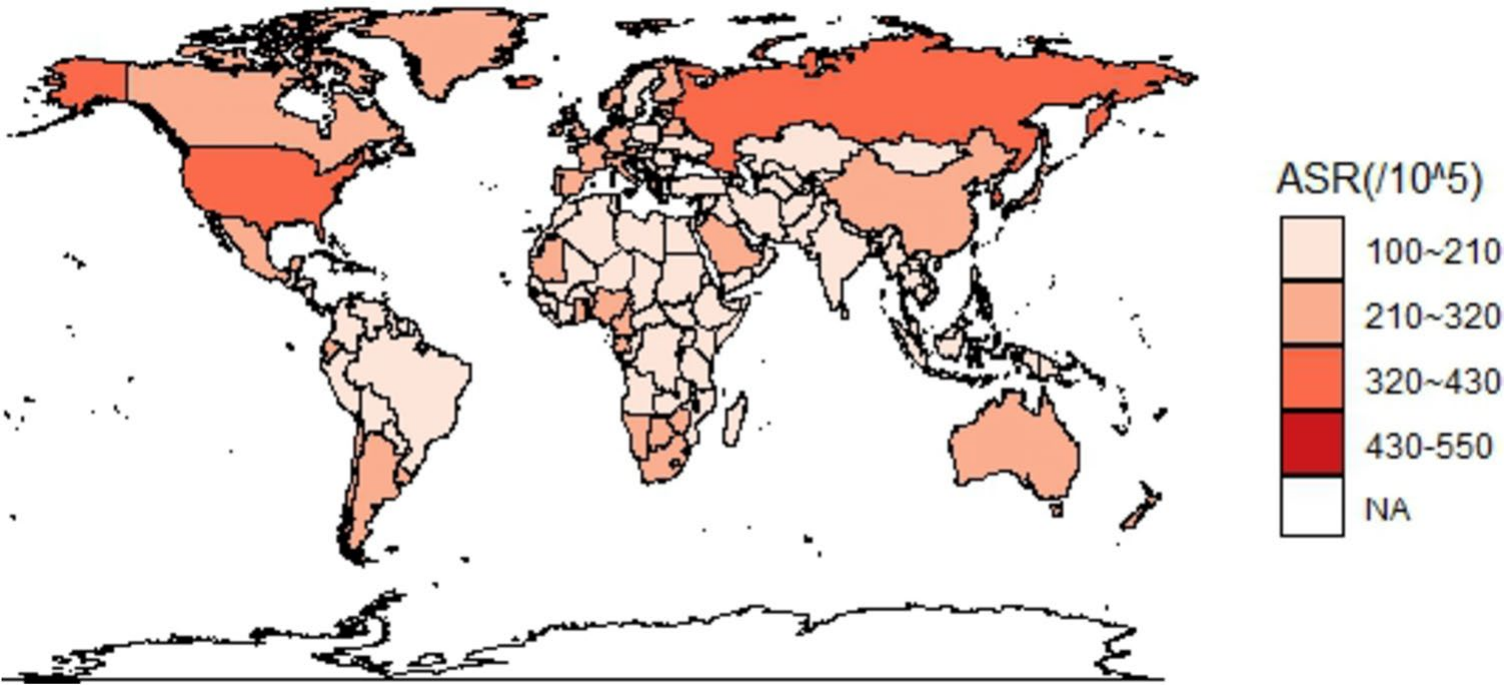
Geographical distribution of DALY rate per 100,000 osteoarthritis worldwide in 2019

Figure 1 shows the geographic distribution of age-standardized incidence rate of osteoarthritis worldwide. The age-standardized incidence rate of osteoarthritis in China (509.84/100,000) was higher than that in Asia (463.34/100,000), Africa (447.83/100,000), and Oceania (396.25/100,000) and lower than that in America (603.22/100,000) and Europe (530/46/100,000). Compared with neighboring Asian countries, the age-standardized incidence rate of osteoarthritis in China was higher than that in North Korea (463.30/100,000) and India (430.04/100,000) and lower than that in South Korea (734.40/100,000) and Japan (645.28/100,000). Figure 2 shows the geographic distribution of the global age-standardized DALY rate of osteoarthritis. The age-standardized DALY rate of osteoarthritis in China (224.78/100,000) was higher than that in Asia (208.03/100,000), Africa (199.55/100,000), and Oceania (166.52/100,000) and lower than that in America (292.14/100,000) and Europe (249.65/100,000). Compared with neighboring countries in Asia, it is higher than North Korea (206.48/100,000) and India (180.21/100,000) and lower than that in South Korea (369.39/100,000) and Japan (304.67/100,000).

### Changes of incidence and DALY rates of osteoarthritis in China in 1990 and 2019

In 2019, the number of cases of osteoarthritis in China was 10,681,311, an increase of 132.66% compared with 1990. In 2019, the number of cases of osteoarthritis in women (6,313,626) was higher than that in men (4,367,685). In 2019, the DALY of osteoarthritis in China was 4,724,885 person-years, which was 159.70% higher than that in 1990. In 2019, DALY (2,827,915) of female osteoarthritis was higher than that of male (1,896,969). In 2019, the incidence of osteoarthritis in China was 750.96/100,000, which was 93.62% higher than that in 1990. In 2019, the incidence of osteoarthritis in women (905.14/100,000) was higher than that in men (602.59/100,000). In 2019, the DALY rate of osteoarthritis in China was 332.19/100,000, which was 116.13% higher than that in 1990. In 2019, the DALY rate of women (405.42/100,000) was higher than that of men (261.71/100,000) (Table 1).

**Table 1:** Changes of incidence and DALY rates of osteoarthritis in China in 1990 and 2019

	Year	Male (95% UI)	Female (95% UI)	Both (95% UI)
Incidence cases	1990	1,917,757 (1,680,160–2,182,079)	2,673,211 (2,359,383–2,993,781)	4,590,967 (4,044,932–5,178,960)
	2019	4,367,685 (3,826,418–4,963,492)	6,313,626 (5,538,885–7,118,134)	10,681,311 (9,375,594–12,079,546)
Changes	(%)	127.75	136.18	132.66
DALY	1990	742,591 (370,777–1,475,682)	1,076,747 (535,442–2,173,533)	1,819,338 (903,102–3,648,574)
	2019	1,896,969 (939,463–3,787,240)	2,827,915 (1,409,989–5,745,787)	4,724,885 (2,347,243–9,536,082)
Changes	(%)	155.45	162.64	159.70
Incidence rate	1990	314.29 (275.35–357.61)	466.13 (411.40–522.03)	387.85 (341.72–437.53)
	2019	602.59 (527.91–684.79)	905.14 (794.07–1020.48)	750.96 (659.16–849.17)
Changes	(%)	91.73	94.18	93.62
DALY rate	1990	121.70 (60.76–241.84))	187.75 (93.36–378.99)	153.70 (76.30–165.03
	2019	261.71 (129.61–522.51)	405.42 (202.14–823.73)	332.19 (165.03–670.45)
Changes	(%)	115.05	115.93	116.13

### Trends of associated risk factors of osteoarthritis from 1990 to 2019

**Fig. 3:**
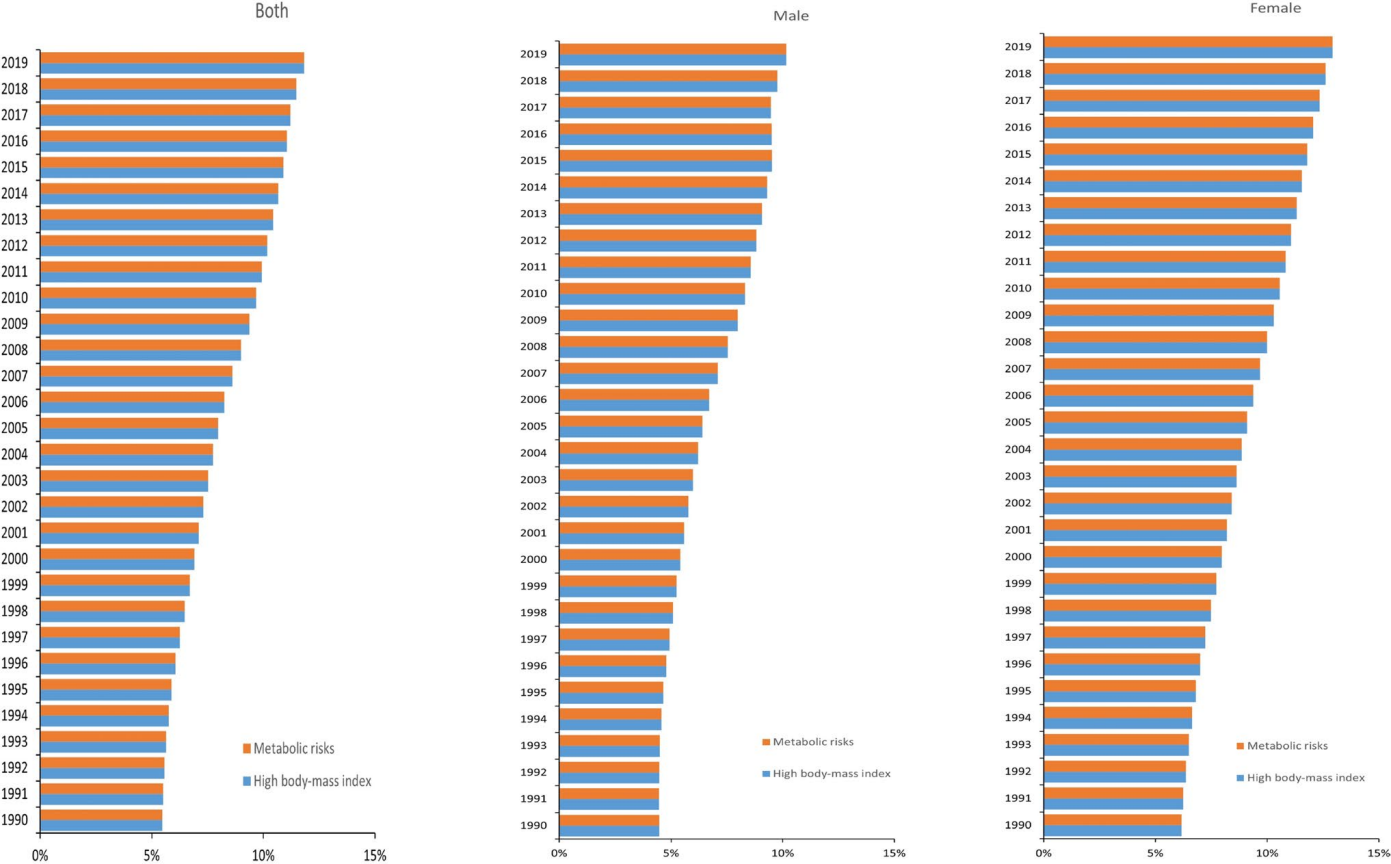
Population attributable proportion (PAF, %) of associated risk factors for osteoarthritis DALY from1990 to 2019

GBD 2019 identified high body mass index as risk factors for osteoarthritis DALY. In China, high body mass index showed a population attributable proportion (PAF) that increased steadily from 1990 to 2019, reaching 5% and 12%, respectively. The PAF for high body mass index in females from 1990 to 2019 demonstrated a steady increase and was always higher than that of men (Fig. 3).

### Disease burden of three types of osteoarthritis from 1990 to 2019

GBD 2019 identified osteoarthritis hip, osteoarthritis knee, and osteoarthritis hand as three types of osteoarthritis. In any year, the incidence rate of three types of osteoarthritis from high to low is osteoarthritis knee, osteoarthritis hand, and osteoarthritis hip. The incidence rate of the male osteoarthritis hip slowly increased from 8.86/100,000 in 1990 to 12.36/100,000 in 2019, and the incidence rate of the female osteoarthritis hip slowly increased from 7.69/100,000 in 1990 to 10.48/100,000 in 2019, and the incidence rate of the osteoarthritis hip in men was greater than that in women in any year. The incidence rate of male osteoarthritis hand fluctuated from 53.18/100,000 in 1990 to 45.13/100,000 in 2019. The incidence of female osteoarthritis hand increased first and then decreased, from 52.13/100,000 in 1990 to 46.08/100,000 in 2019. The incidence of male osteoarthritis knee declined first and then increased, from 287.38/100000 in 1990 to 305.03/100,000 in 2019. The incidence rate of female osteoarthritis knee also showed a trend of decline and increase, from 471.81/100,000 in 1990 to 500.03/100,000 in 2019. And the incidence rate of osteoarthritis knee in women is higher than that in men in any year.

**Fig. 4:**
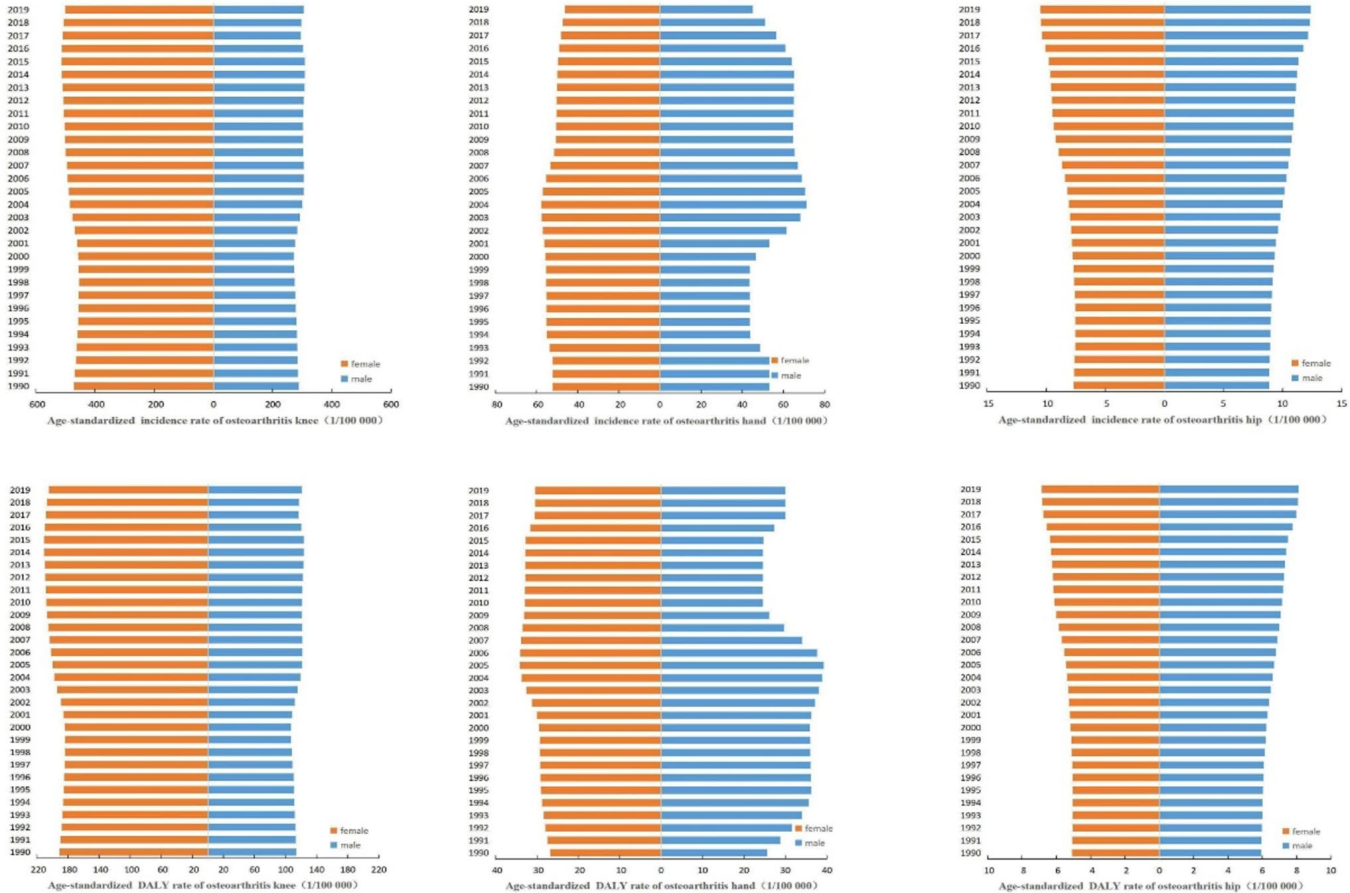
Disease burden of three types of osteoarthritis from 1990 to 2019

In any year, the DALY rate of three types of osteoarthritis from high to low is osteoarthritis knee, osteoarthritis hand, and osteoarthritis hip. The male osteoarthritis hip DALY rate slowly increased from 5.93/100,000 in 1990 to 8.12/100,000 in 2019, and the female osteoarthritis hip DALY rate slowly increased from 5.09/100,000 in 1990 to 6.85/100,000 in 2019. Moreover, the osteoarthritis hip DALY rate of males is higher than that of females in any year. The DALY rate of osteoarthritis hand fluctuates between males and females. The DALY rate of osteoarthritis hand in males increased from 25.56/100,000 in 1990 to 29.95/100,000 in 2019, and the DALY rate of osteoarthritis hand in females increased from 26.79/100,000 in 1990 to 30.42/100,000 in 2019. The DALY of male osteoarthritis knee first decreased and then increased, from 113.38/100,000 in 1990 to 120.68/100,000 in 2019. The DALY rate of female osteoarthritis knee also showed a decreasing and then increasing trend, from 190.73/100,000 in 1990 to 204.60/100,000 in 2019. And the incidence rate of osteoarthritis knee in women is higher than that in men in any year (Fig. 4).

### Age–period–cohort analysis for osteoarthritis incidence and DALY rates in China

**Fig. 5:**
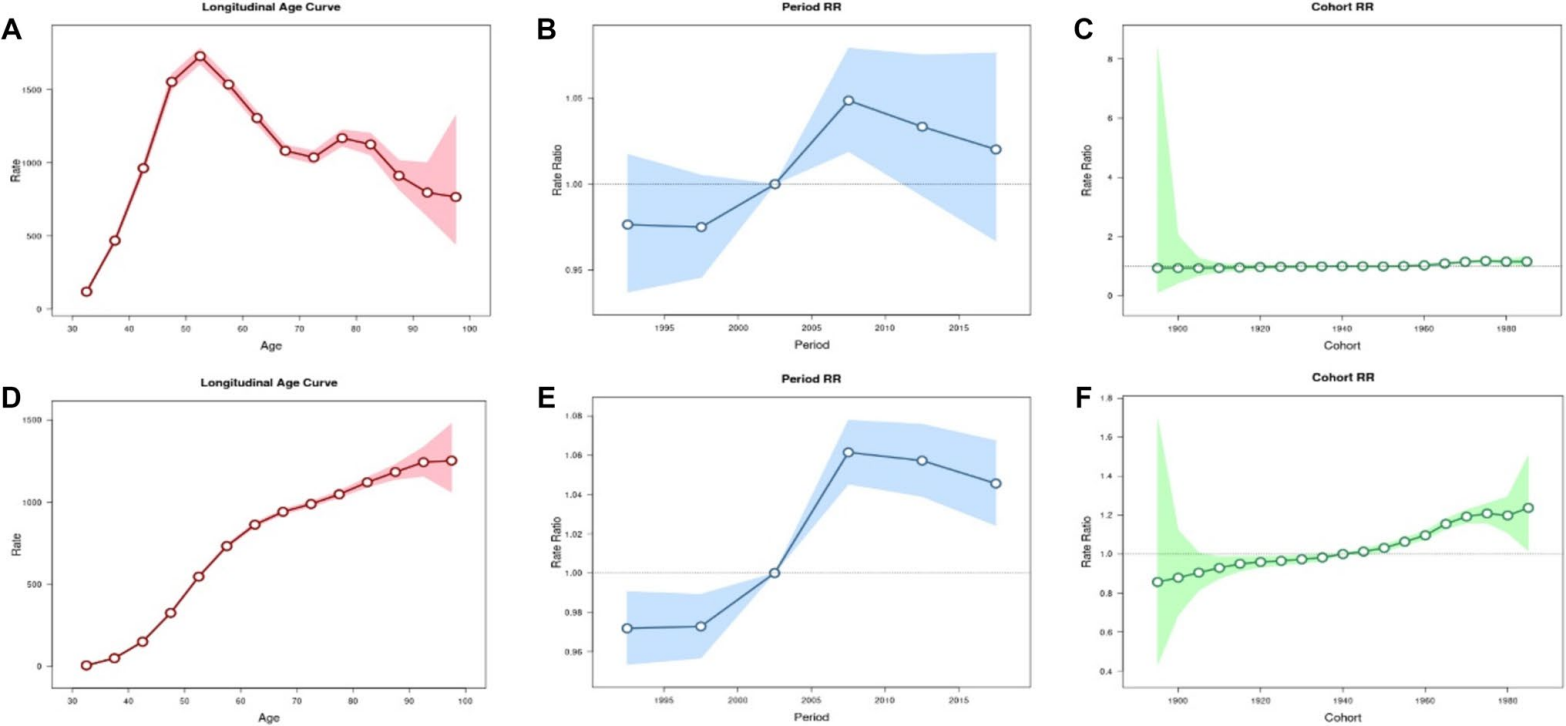
Age–period–cohort analysis for incidence and DALY rates of osteoarthritis in China. **A** Age effect for incidence rate. **B** Period effect for incidence rate. **C** Cohort effect for incidence rate. **D** Age effect for DALY rate. **E** Period effect for DALY rate. **F** Cohort effect for DALY rate

Table 2 shows that the net drift values of osteoarthritis were 0.252% (95% CI − 0.075, 0.580) per year for incidence and 0.386% (95% CI 0.275, 0.498) per year for DALY. When the period and cohort effects were controlled, the incidence rate of osteoarthritis in China shows a trend of increasing first and then decreasing with age, the DALY rate of osteoarthritis in China increased with age. For the period effect, we found that the period *RR* of osteoarthritis incidence and DALY rates kept increasing in the cohort born before 2005–2009, and then, it was gradually reduced by year of birth in the cohort born after 2005–2009. As for cohort effect, the cohort *RR* of incidence rate of osteoarthritis almost has no change, while cohort *RR* fluctuates around *RR* = 1; the cohort *RR* of DALY rate of osteoarthritis kept increasing from 1990 to 2019 (Fig. 5).

**Table 2:** Statistical parameters of osteoarthritis in age–period–cohort models

Type	Net drift (% per years; 95% CI)	*P* value		
		All local drifts = net drift	All cohort deviations = 0	All period deviations = 0
Incidence	0.252 (− 0.075 to 0.580)	< 0.001	< 0.001	< 0.001
DALY	0.386(0.275 to 0.498)	< 0.001	< 0.001	< 0.001

## Discussion

Osteoarthritis is one of the main causes of disability in the world. It is reported that its incidence is increasing year by year [16, 17], which brings a huge burden on the health care system due to its high disability rate. Many papers reported the global burden of osteoarthritis [18, 19]. In this study, we put forward the most comprehensive and up-to-date osteoarthritis burden and provide the age, period, and cohort effects on the long-term trends of osteoarthritis incidence and DALY in China from 1990 to 2019.

This study shows that with neighboring Asian countries, the age-standardized incidence and DALY rates of osteoarthritis in China were higher than that in North Korea and India, lower than that in South Korea, and Japan. It shows that the disease burden of osteoarthritis is related to the level of national economic development. The higher the level of economic development, the higher the disease burden of osteoarthritis. Similar results were also reported in previous studies [20, 21], and an increasing life expectancy is a non-negligible explanation for this result. In 2019, the number of cases and incidence rate of osteoarthritis in China was 10,681,311 and 750.96/100,000, respectively. The DALY and DALY rate of osteoarthritis were 4,724,885 person-years and 332.19/100,000, respectively. Compared with 1990, all indicators have increased. Similar results were also reported in previous studies [22, 23], with the suggestion that the burden of osteoarthritis would increase over time and were predicted to be one of the leading causes of disability rate, globally. Due to the decrease of exercise, the probability of secondary cardiovascular and cerebrovascular diseases and metabolic diseases in patients with osteoarthritis is greatly increased, and the risks of diabetes, heart failure, and ischemic heart disease are 1.41 times, 1.4 times, and 1.33 times higher than those in the general population, respectively [24]. Thus, it is essential that prevention measures, management, and treatment of osteoarthritis are given priority. This study also found that the incidence and DALY rates of women with osteoarthritis were higher than those of men; explanation for this difference might be the generally stronger joint support in men, as they mostly have more muscle bulk and stronger ligaments. These differences can lead to more traumatic fractures and the imposition of higher joint stress in women, when compared to men [25].

Pain and disability are the most common complaints among patients with osteoarthritis. There are multiple pain-relieving treatments suggested for osteoarthritis; the long-term effects of these treatments remain controversial [26]. Modifiable risk factors are the most crucial targets of osteoarthritis prevention and include high BMI and knee injuries [27], which could be prevented. Previous research has shown that the most supported preventative methods are BMI reductions and avoiding continuous chronic joint stress [28, 29]. More recent studies reported that the prevalence of adult obesity has doubled in more than 70 countries during the past three decades [30]; the obesity rate of Chinese adults has also shown an increasing trend in recent years [31]. This study found that high body mass index with the PAF increases steadily from 1990 to 2019. Therefore, the disease burden of osteoarthritis in China is likely to continue to increase in the future. The knee was the joint most affected by osteoarthritis, followed by the hand and hip. From 1990 to 2019, trends in osteoarthritis incidence rate differed by anatomic site, except that the changes in osteoarthritis hand are decreasing, and the changes in osteoarthritis hip and osteoarthritis knee is increasing.

The age–period–cohort model can further study long-term trends in incidence and DALY rates of osteoarthritis and provide basic theoretical for the health administrative department to formulate prevention and treatment strategies for osteoarthritis [32]. The age effect is the risk of disease caused by age factors. The results of this study show that the incidence rate of osteoarthritis increases first and then decreases with age, while the DALY rate of osteoarthritis increased with age. Osteoarthritis may seem to be inevitable with increased age [33]; it can be halted and/or postponed with the adoption of the previously mentioned lifestyle changes. These interventions are most effective if they are implemented in the early years of childhood [34]. Period effect refers to the improvement of public health intervention measures, health education policies, and treatment methods in a certain period, which will have an impact on the period effect of onset and death of osteoarthritis [35]. The results of the period effect in this study show that the period *RR* of osteoarthritis incidence and DALY rates kept increasing in the cohort born before 2005–2009, and then, it was gradually reduced by year of birth in the cohort born after 2005–2009. The possible reason is that after 2005–2009, through extensive health education [36]and the emergence of new treatment methods [37–39] in recent years, the incidence and DALY rate of osteoarthritis will be reduced. The cohort effect refers to the risk of exposure to different levels of social, natural, and environmental factors, depending on the age of birth of the population. The results of the cohort effect in this study show that the cohort *RR* of incidence rate of osteoarthritis has almost no change, while the cohort *RR* of DALY rate of osteoarthritis kept increasing from 1990 to 2019. Compared with the cohort born in 1940 (*RR* = 1), the cohort born in 1980 has the highest DALY risk, with *RR* value of 1.24, which indicates that young patients with osteoarthritis have higher DALY risk than middle-aged and elderly patients.

This study has several limitations. First, the osteoarthritis data were extracted from GBD 2019, which had varied data sources including surveillance system data and individual-level survey data, implying that the selection bias could affect the certainty of osteoarthritis burden estimates. Second, due to the unavailability of provincial data, this study did not have a geographic description of the burden of osteoarthritis. Lastly, the age–period–cohort model was analyzed at the population level, so that it may be subject to ecological fallacy.

## Conclusion

Osteoarthritis is a major public health challenge; its burden is increasing in China, especially among women. This trend is expected to continue as the aging of China population is rising. Attempts to mitigate the future burden of osteoarthritis require better awareness, especially of the risk factors, and early diagnosis and treatment of osteoarthritis together with the improvement of healthcare infrastructure for managing the increasing number of patients with osteoarthritis. Continuing to expand osteoarthritis population-based data collection at the national level is essential to monitor the disease burden and to further deal with the need for better care.

## Data Availability

Data are available on request.

## Declarations

### Generative AI in scientific writing

The authors are ultimately responsible and accountable for the contents of the work.

### Submission declaration and verification

This article is not under consideration for publication elsewhere.

## Abbreviations

*DALY*: Disability-adjusted life years
*GBD*: Global Burden of Disease study
*RR*: Rate ratio
*APC*: Age–period–cohort
*PAF*: Population attributable proportion
